# Salivary Biomarkers to Differentiate between *Streptococcus pneumoniae* and Influenza A Virus-Related Pneumonia in Children

**DOI:** 10.3390/diagnostics13081468

**Published:** 2023-04-18

**Authors:** Kuo-Shu Tang, Chih-Min Tsai, Ming-Chou Cheng, Ying-Hsien Huang, Chih-Hao Chang, Hong-Ren Yu

**Affiliations:** 1 Department of Pediatrics, Kaohsiung Chang Gung Memorial Hospital, Kaohsiung 83301, Taiwan; tang1004@cgmh.org.tw (K.-S.T.); tcmnor@cgmh.org.tw (C.-M.T.); 8802043@cgmh.org.tw (M.-C.C.); yhhuang123@yahoo.com.tw (Y.-H.H.); 2 Graduate Institute of Clinical Medical Sciences, Chang Gung University College of Medicine, Taoyuan City 33302, Taiwan; 3 Department of Respiratory Therapy, Kaohsiung Chang Gung Memorial Hospital, Kaohsiung 83301, Taiwan

**Keywords:** children, pneumonia, *Streptococcus pneumoniae*, influenza A, saliva, biomarkers

## Abstract

Community-acquired pneumonia (CAP) is common among children and can be fatal in certain conditions. In children, CAP can be caused by viral or bacterial infections. Identification of pathogens can help select appropriate therapeutic strategies. Salivary analysis may be a potential diagnostic tool because it is noninvasive, patient-friendly, and easy to perform in children. A prospective study was conducted in children with pneumonia admitted to a hospital. Salivary samples from patients with definite *Streptococcus pneumoniae* and influenza A strains were used for gel-free (isobaric tag for relative and absolute quantitation (iTRAQ)) proteomics. No statistically significant difference was detected in salivary CRP levels between *Streptococcus pneumoniae* and influenza A pneumonia in children. Several potential salivary biomarkers were identified using gel-free iTRAQ proteomics to differentiate pneumonia from *Streptococcus pneumoniae* or influenza A virus infections in pediatric patients. ELISA validated that *Streptococcus pneumoniae* group has a higher abundance of salivary alpha 1-antichymotrypsin than those in the influenza A group. Whether these salivary biomarkers can be used to distinguish other bacteria from viral pneumonia requires further verification.

## 1. Introduction

The global number of deaths among children under 5 years old substantially decreased over the past two decades from 9.65 million in 2000 to 5.05 million in 2019; however, lower respiratory infections still account for 13.3% of the deaths [1]. Community-acquired pneumonia (CAP) in children can be caused by various pathogens, including viruses and bacteria. *Streptococcus pneumoniae*, *Staphylococcus aureus*, *Mycoplasma pneumoniae*, and *Haemophilus influenzae* type b (Hib) are the most common pathogens causing bacterial CAP in children [2,3,4,5]. Respiratory syncytial viruses, influenza viruses, adenoviruses, and rhinoviruses are common causes of community pneumonia in children [6]. The identification of pathogens can help select appropriate therapeutic strategies and reduce the use of unnecessary antibiotics.

In children with purulent bacterial pneumonia, the initial clinical manifestations may be similar to those of viral infections, such as cough/rhinorrhea, low titers of inflammatory markers, and chest radiographs showing interstitial infiltration. Children with atypical bacterial pneumonia may also show clinical presentations and laboratory and imaging findings similar to those with viral pneumonia [3]. Nonspecific biomarkers, such as C-reactive protein (CRP) and procalcitonin, are valuable for assessing the severity of the disease and response to treatment in patients with pneumonia; however, it remains a challenge to distinguish between bacterial and viral infections [3,7,8,9]. Currently, no clinical algorithm can clearly distinguish the etiology of pneumonia.

Sputum culture or polymerase chain reaction (PCR) was used to identify pathogens. However, sputum samples from children are often contaminated with saliva, and direct pathogenic evidence of pulmonary infection in children often requires invasive procedures to obtain lower respiratory tract specimens for culture and other laboratory tests. As children are less likely to cooperate, invasive procedures are usually reserved for seriously ill patients or those with severe comorbidities who are deteriorating despite empirical therapy. In most cases, testing for pneumonia pathogens is performed from alternative sources, such as the nasopharynx or blood, which are not necessarily representative of the causative agents of pneumonia.

In children, saliva collection is easier and more noninvasive than blood sampling. Saliva analysis has the potential to become an emerging diagnostic tool for pediatric patients [10,11,12,13]. In a previous study, we showed that salivary C-reactive protein (CRP) levels were highly correlated with serum CRP levels in pediatric patients with pneumonia [14]. This study aimed to determine whether salivary CRP levels could help distinguish between bacterial and viral pneumonia in children. We also aimed to identify potential salivary biomarker levels that could assist in differentiating bacterial or viral infections in pediatric patients with pneumonia.

## 2. Materials and Methods

### 2.1. Study Design and Population

This prospective study was conducted from 1 January 2018 to 31 January 2019, at the Pediatric Ward, Kaohsiung Chang Gung Hospital, Kaohsiung, Taiwan. Children and adolescents aged 2–17 years old, who were diagnosed with CAP, were included in this study. CAP was defined as a previously healthy child presenting signs and symptoms of pneumonia and consolidation on chest radiography owing to a community-acquired infection. Patients with cerebral palsy, immunodeficiency, malignancies, or other chronic lung diseases were excluded. Patients underwent routine analysis of serum CRP levels and peripheral complete blood counts with differential white blood cell counts on admission. Saliva and serum samples were collected on the same day as the biomarker studies. Patients were also investigated for pathogens, including viruses (e.g., influenza and adenovirus antigen rapid test, and viral isolates), *Mycoplasma pneumoniae* serum IgM ELISA [15,16,17] (ImmunoWELL; Ben-Bio, San Diego, CA, USA), blood cultures, and urine antigen testing for *Streptococcus pneumoniae*. Written informed consent was obtained from the parents or guardians of the participants. This study was approved by the Institutional Review Board of Chang Gung Medical Foundation (IRB number: 201801539A3C501).

### 2.2. Saliva Collection

Saliva samples were collected using SalivaBio Pediatric Swabs (Salimetrics, State College, PA, USA), as previously reported [14]. After collection, the saliva samples were centrifuged at 4000 rpm for 10 min. The supernatant was stored at −80 °C until further analysis.

### 2.3. Gel-Free Isobaric Tags for Relative and Absolute Quantitation (iTRAQ) Proteomics

In this study, isobaric tagging for relative and absolute quantification (iTRAQ) gel-free proteomics was used to identify salivary protein biomarkers. To screen salivary protein biomarkers, we randomly selected saliva samples from age-matched *Streptococcus pneumoniae* (*S. pneumoniae*) infection and *influenza A*-associated pneumonia cases for proteomic analysis. The selected saliva samples were subjected to high-abundance protein depletion using Pierce Top 12 Abundant Protein Depletion Spin Columns (85,165; Thermo Fisher Scientific, Waltham, MA, USA). Salivary samples from each of the three subjects were combined to obtain a combined pneumonia sample from *Streptococcus pneumoniae* and Type A influenza. Salivary samples were labeled and prepared using the iTRAQ Reagents Multiplex Kit (435,2135, AB SCIEX, Foster City, CA, USA). After standard quality control inspection, the labeled salivary samples were analyzed using LC/Q-Exactive Orbitrap MS (Thermo) and interpreted with Proteome Discoverer v2.4 (Thermo) referring to the MASCOT 2.5 database to obtain the relative abundance of proteins.

### 2.4. Salivary Biomarker Determination

Serum CRP levels were quantified using a Cobas 6000 analyzer (Roche, Mannheim, Germany). Salivary biomarkers were measured using commercial ELISA kits: CRP (Salimetrics^®^, State College, PA, USA), Serpin Family A Member 3 (SERPINA3; Alpha 1-Antichymotrypsin) (MyBiosource, LLC, San Diego, CA, USA), and complement factor D (CFD) (R&D Systems^®^, Minneapolis, MN, USA).

### 2.5. Statistical Analysis

All statistical analyses were performed using the IBM SPSS Statistics version 22 (IBM SPSS Statistics for Windows, Version 22.0. Armonk, NY, USA: IBM Corp). The statistical significance level for all tests was *p* < 0.05. Continuous variables are expressed as mean ± standard error of the mean. Categorical data were analyzed using the chi-squared test. Student’s *t*-test, or a non-parametric method was used to compare the results of continuous variables. Statistical correlations were determined using the Spearman’s rank correlation test.

## 3. Results

### 3.1. Patients

During the study period, 88 pediatric patients diagnosed with pneumonia were enrolled in this study, consisting of 41 boys and 39 girls with a mean age of 5.27 ± 0.62 years. Among them, eight cases were identified as *Streptococcus pneumoniae* infection by urine antigen, two cases were identified as Group A streptococcus antigen-positive by throat swab, seven cases were identified as influenza A virus by virus isolation, one case was adenovirus antigen-positive, and parainfluenza virus was isolated from one case by throat swab. Another 55 cases were positive for a single serum *Mycoplasma pneumoniae* IgM, and no other specific pathogen was identified.

### 3.2. Salivary and Serum CRP Levels Were Highly Correlated

**Figure 1:**
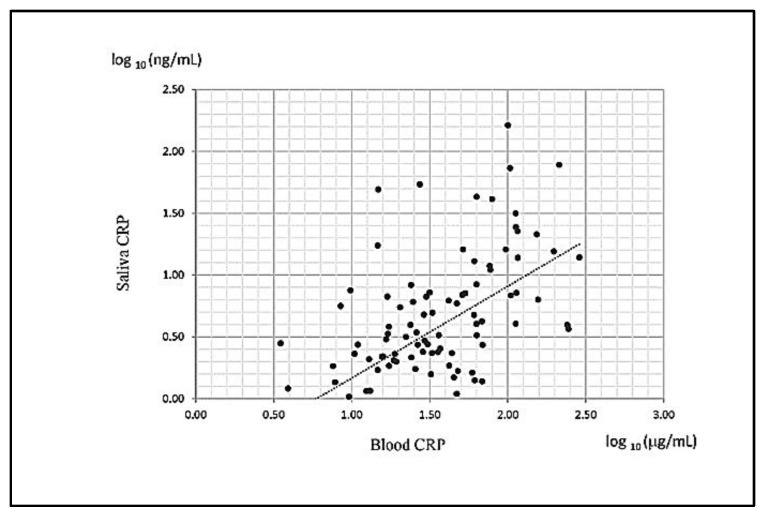
Scatter diagram of the serum and corresponding salivary CRP levels of children with pneumonia.

In a previous study, we reported that the salivary CRP levels in children with pneumonia were highly correlated with the serum CRP levels. In the present study, we verified the correlation between serum and salivary CRP levels. Among the 88 enrolled patients, a strong positive correlation was detected between salivary and serum CRP levels (r = 0.616; *p* < 0.001) (Figure 1).

### 3.3. Salivary CRP Levels Did Not Distinguish Whether the Pathogen Causing Pneumonia in Children was Streptococcus pneumoniae or Influenza A

**Figure 2:**
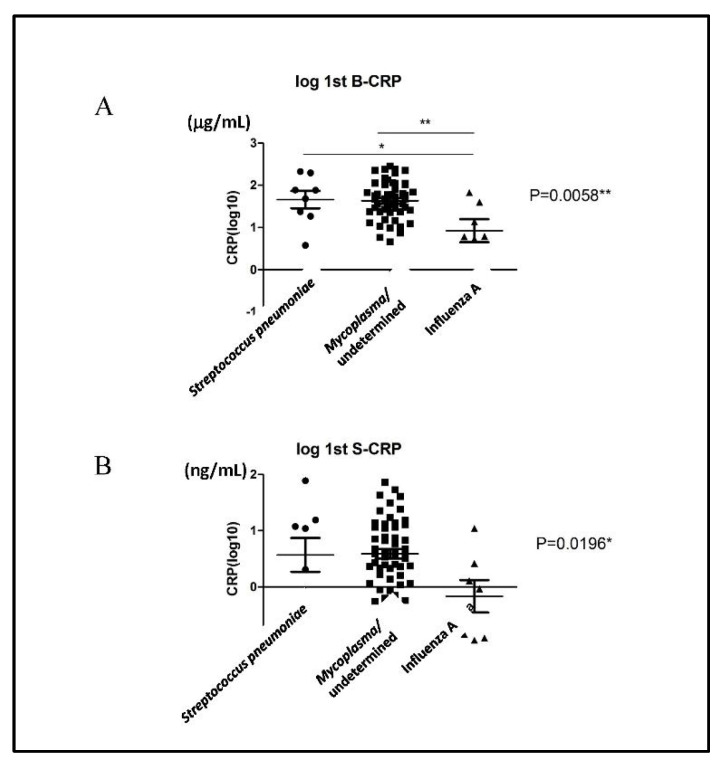
The (**A**) serum and (**B**) salivary CRP levels in *Streptococcus pneumoniae*, Mycoplasma pneumoniae/undetermined, and influenza A virus groups. * *p* < 0.05; ** *p* < 0.01.

Serum and salivary CRP levels were further compared in eight cases identified as *Streptococcus pneumoniae* infection by urine antigen, seven cases identified as influenza A virus by virus isolation, and 55 cases positive for serum Mycoplasma pneumoniae IgM with no other specific pathogen. Mean salivary and serum CRP levels in the three groups were compared. The *Streptococcus pneumoniae* group and Mycoplasma pneumoniae/undetermined group had higher serum CRP levels than those of the influenza A group (Figure 2A). Salivary CRP levels could not be used to distinguish *Streptococcus pneumoniae* and influenza A infections (Figure 2B). Because determining the pathogen of pneumonia is helpful for determining therapeutic strategies in the clinic, we investigated potential biomarkers from saliva to help distinguish *Streptococcus pneumoniae* from influenza A virus infection.

### 3.4. Identification of the Differential Salivary Biomarkers from Children with Streptococcus pneumoniae and Influenza a Virus Infection by Gel-Free Isobaric Tag for Relative and Absolute Quantitation (iTRAQ) Proteomics

**Figure 3:**
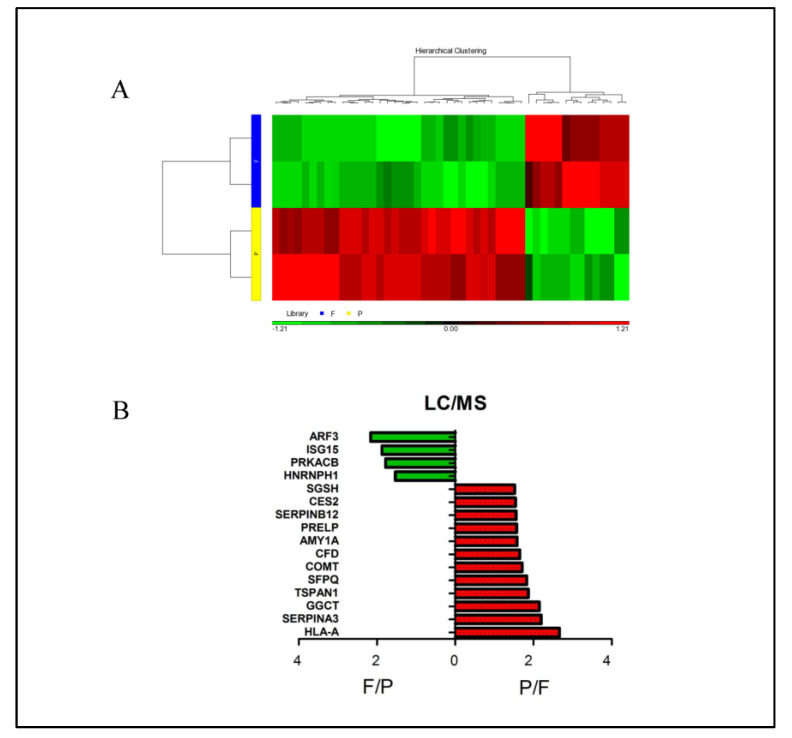
Differential salivary proteins between *Streptococcus pneumoniae* (P) and influenza A virus (F) groups. (**A**) Heat map was obtained by comparing the 47 differentially expressed proteins identified by gel-free iTRAQ proteomics analysis. The picture is colored according to the abundance: green and red represent relatively low and high abundance, respectively. (**B**) Ranking of proteins by abundance more than 1.5-fold, 4 proteins were more abundant in the influenza A group, and 12 proteins were more abundant in the *Streptococcus pneumoniae* group. Abbreviations: ARF3, ADP-ribosylation factor 3; ISG15, Interferon-stimulated gene 15; PRKACB, cAMP-dependent protein kinase catalytic subunit beta; HNRNPH1, heterogeneous nuclear ribonucleoprotein H1; SGSH, n-sulphoglucosamine sulphohydrolase; CES2, carboxylesterase 2; SERPINB12, serpin family B member 12; PRELP, prolargin, AMY1A, alpha-amylase 1; CFD, complement factor D, COMT, catechol-O-methyltransferase; SFPQ, splicing factor, proline- and glutamine-rich; TSPAN1, tetraspanin 1; GGCT, gamma-glutamylcyclotransferase, SERPINA3, alpha 1-antichymotrypsin, and HLA-A, human leukocyte antigens-A.

Further studies were conducted to determine differential salivary biomarkers in children with *Streptococcus pneumoniae* and influenza A virus infections. Children with a single *Mycoplasma pneumoniae* IgM positivity were not included for further study because of the high false-positive and false-negative rates [15,18,19]. We randomly selected two saliva samples from children with Streptococcus pneumonia and influenza A virus pneumonia for the gel-free iTRAQ proteomic study. Based on both parameters, protein and peptide identifications with false determinate rate (FDR) < 0.01 and the protein identified with at least two unique peptides (UP) ≥ 2, we identified 1169 proteins in the saliva samples of *Streptococcus pneumoniae* and influenza A groups. After comparing the protein abundances with those of Partek, 48 proteins were determined to be differentially abundant (variation > 1.2-fold) between the *Streptococcus pneumoniae* and influenza A groups (Supplementary Table S1). Both types of salivary samples were well-separated based on the abundance profiles of the 47 proteins (Figure 3A). When the variation was >1.5 fold, 12 and 4 proteins were more abundant in the saliva samples from the *Streptococcus pneumoniae* and influenza A groups, respectively, than those in the other groups (Figure 3B). Saliva samples from the influenza A group were more abundant in ADP-ribosylation factor 3 (ARF3), interferon-stimulated gene 15 (ISG15), cAMP-dependent protein kinase catalytic subunit beta (PRKACB), and heterogeneous nuclear ribonucleoprotein H1 (HNRNPH1) than those from the *Streptococcus pneumoniae* group. The proteins with higher abundance in saliva samples from the *Streptococcus pneumoniae* group than those from the influenza A group were n-sulphoglucosamine sulphohydrolase (SGSH), carboxylesterase 2 (CES2), serpin family B member 12 (SERPINB12), prolargin (PRELP), alpha-amylase 1 (AMY1A), complement factor D (CFD), catechol-O-methyltransferase (COMT), splicing factor, proline- and glutamine-rich (SFPQ), tetraspanin 1 (TSPAN1), gamma-glutamylcyclotransferase (GGCT), alpha 1-antichymotrypsin (SERPINA3), and human leukocyte antigens (HLA)-A.

### 3.5. Different Pathways Were Enriched by Specific Salivary Proteins from Children with Streptococcus pneumoniae and Influenza A Infections

To investigate the differential functions of salivary proteins related to *Streptococcus pneumoniae* and influenza A infections, KEGG pathway enrichment analyses were conducted. The top five most significant pathways related to *Streptococcus pneumoniae* were valine, leucine, and isoleucine biosynthesis; salivary secretion; complement and coagulation cascades; amoebiasis; and lysosomes (Table 1). Vasopressin-regulated water reabsorption, amphetamine addiction, GABAergic synapses, long-term potentiation, and inflammatory mediator regulation of TRP channel pathways were the five most significant pathways related to influenza A infection (Table 2). To highlight the roles of differentially abundant proteins within the enriched pathways, we plotted the enriched pathways. The top 5 most significant pathways in Table 1 and Table 2 were provided in Supplementary Figures S1–S10. The genes/proteins highlighted with red and green boxes denoted up- and down-regulated, respectively.

### 3.6. Validation of Differential Proteins in Saliva Samples between the Streptococcus pneumoniae and Influenza A Groups

**Figure 4:**
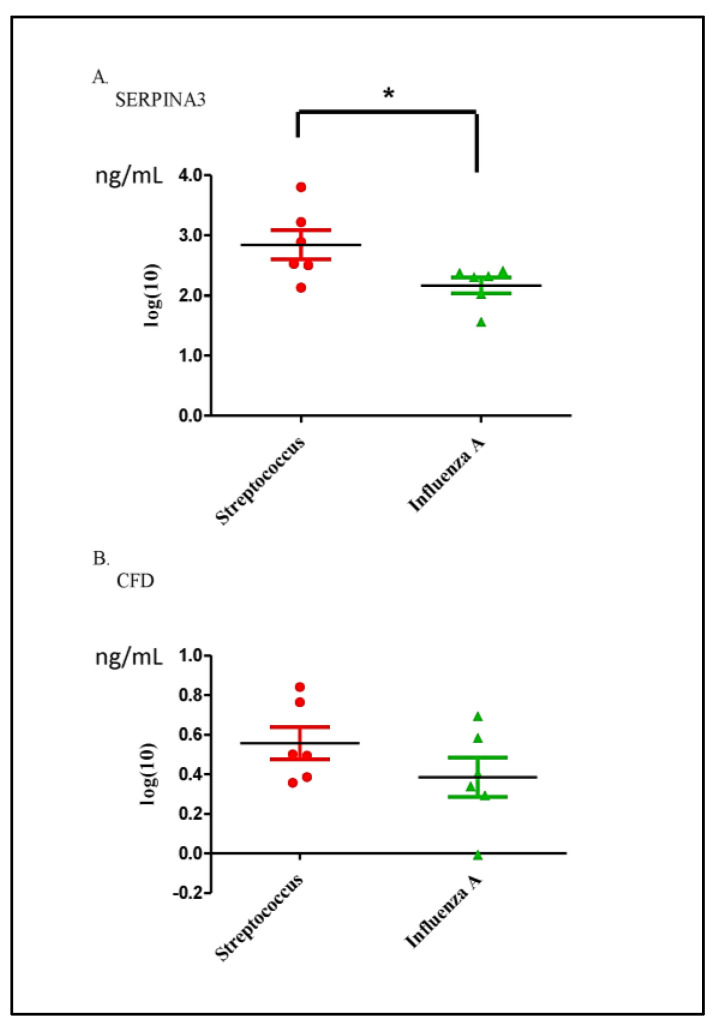
(**A**) Salivary alpha 1-antichymotrypsin (SERPINA3) and (**B**) complement factor D (CFD) levels in *Streptococcus pneumoniae* and influenza A virus groups. The SERPINA3 and CFD levels were determined with ELISA and compared with a non-parametric method. Due to low concentration, data are transformed to Log10 for presentation. * *p* < 0.05.

Because of their availability, SERPINA3 and CFD ELISA were chosen to validate the differences in salivary between S. pneumoniae and influenza A virus infections. Although 8 cases of *Streptococcus pneumoniae* and 7 cases of influenza A virus associated pneumonia were initially enrolled, after CRP detection and proteomic analysis, only 6 cases in each group were enough for ELISA determination (Figure 4). Compared with those of the influenza group, the salivary samples of children with *Streptococcus pneumoniae* infection had significantly higher SERPINA3 levels. In contrast, the salivary samples from the influenza A group had higher CFD than those from the *Streptococcus pneumoniae* group, but the difference was not statistically significant.

## 4. Discussion

Epidemiological factors, clinical manifestations in sick children, and chest radiographic patterns suggest the causative agent; however, they cannot be used as criteria to reliably differentiate between bacterial and atypical bacterial or viral infections in childhood pneumonia. Although the development of multiple nucleic acid detection technologies, such as polymerase chain reaction, improved the diagnosis of CAP etiology, a fundamental dilemma still exists in the identification of pneumonia pathogens in children. First, ethical and technical issues limit clinicians from directly obtaining specimens from the lower respiratory tract of sick children [20]. Second, the use of antibiotics will affect the sensitivity of microbial cultures and may also affect the concentration of inflammatory markers [21]. Third, owing to the dynamic nature of the infection, the results of testing specimens at different stages of the disease course are likely to vary. Fourth, medical resources differ in different regions, and multiple nucleic acid detection technologies are readily available.

Serum biomarkers have long been important in the diagnosis and treatment of infectious diseases [22]. Recently, after reviewing 31 studies of 23 biomarkers, a meta-analysis of the ability of biomarkers to identify bacterial pneumonia concluded that although serum CRP and procalcitonin (PCT) perform better than white blood cell count or erythrocyte sedimentation rate, the sensitivity and specificity of these markers are between 60% and 70%, which is still unsatisfactory [23]. Developed biomarker approaches, including transcriptomics, proteomics, and metabolomics, are emerging as potential methods for etiological profiling. Tsalik et al. (2016) developed classifiers for acute respiratory bacterial and viral infections in adults using peripheral whole-blood transcriptomics with an overall accuracy of up to 87% and passing external validation (area under the curve (AUC) > 0.90) [24]. Another study conducted a multi-cohort meta-analysis of blood transcriptome data available in public databases and identified three genes, *BAFT*, *ISG15*, and *DNMT1*, that could distinguish between viral and bacterial infections with high accuracy (training set: AUC: 0.86, sensitivity: 0.81, and specificity: 0.87) [25]. Oved et al. (2015) developed a blood multiprotein signature composed of a TNF-related apoptosis-inducing ligand (TRAIL), interferon gamma-induced protein 10 (IP-10), and serum CRP using a proteomics method. This multi-protein signature provided high accuracy and stability in distinguishing bacteria from patients with viral infection (sensitivity, 87.8–99.8% and specificity, 85.6–94.0%) [26,27,28,29]. However, all these proteosome-related studies were reported by a research team in Israel; thus, cross-ethnic validations are required.

Recently, saliva gradually gained recognition in specific fields as a diagnostic alternative to blood collection [30,31,32,33,34]. Because saliva collection is simple, noninvasive, inexpensive, and requires relatively simple instructions, it is particularly suitable for use in pediatric patients. CRP, an acute-phase response protein synthesized by the liver in response to elevated cytokine levels, was studied as a sensitive marker of inflammatory conditions [35]. In our previous study, we found that salivary CRP levels were positively correlated with serum CRP levels, which is supported by previous reports [14,30,36]. However, in this preliminary study, we failed to find that salivary CRP was helpful in distinguishing *Streptococcus pneumoniae* from influenza A virus-related CAPs. Whether salivary CRP levels can help distinguish other bacterial and viral CAPs requires further verification.

ARF3, ISG15, and HNRNPH1 were more abundant in the salivary samples of the influenza A group than in those of *Streptococcus pneumoniae* group. ADP ribosylation factor (ARF) is a small guanosine triphosphate (GTP)-binding protein that participates in the formation of coated membranous vesicles originating from different organelles and is required for vesicular transport [37]. ARF3 is involved in the replication of poliovirus and enterovirus 71 [37,38]. ISG15 plays a central role in host antiviral response [39]. In the case of influenza A virus infection, ISG-15 affects viral genome replication [40]. HNRNPH1 is an RNA-binding protein that forms complexes with heterogeneous nuclear RNA and participates in RNA editing, modification, and stability [41]. HNRNPH1 is involved in the replication of HIV-1 and Newcastle disease virus [42,43]. However, their role in saliva during influenza A infection remains unclear. Alpha-1-antichymotrypsin (SERPINA3) is a serine protease inhibitor, and its plasma concentration can rapidly increase within a short period after tissue damage. In addition to hepatocytes, bronchial epithelial cells, macrophages, and T-cells also produce high concentrations of α1-antichymotrypsin upon stimulation [44,45,46]. Alpha-1-antichymotrypsin can inhibit chemotaxis, superoxide synthesis, and platelet-activating factor synthesis in neutrophils [44]. Alpha-1-antichymotrypsin is, therefore, considered a key player in the inflammatory processes that protect host tissues from proteolytic and oxidative damage [47]. Because α1-antichymotrypsin is closely related to neutrophil activation, this could explain its higher levels in saliva during *Streptococcus pneumoniae* infection than those during influenza A virus infection.

Our study had several limitations. First, to clarify the aim of the study, only cases of pneumonia with identified pathogen(s) were selected for salivary proteomic analyses. Among the patients with identified pathogens, only *Streptococcus pneumoniae* and type A influenza had the highest numbers. Second, we did not enroll patients with pneumonia diagnosed with *Mycoplasma pneumoniae* infection because *M. pneumoniae* IgM may persist for several months after infection [15,18]. Third, owing to the rise in COVID-19 infections, the collection of saliva samples was interrupted based on the requirements of hospital infection control. Consequently, only a limited number of patients were included in the study. Further verification experiments on whether the identified markers could distinguish *Streptococcus pneumoniae* and influenza A in actual detection are necessary. Several differentially expressed proteins needed to be verified. Furthermore, there is an urgent need to elucidate the mechanisms that transmit systemic diseases to the saliva.

## 5. Conclusions

This study showed that the salivary CRP level was not helpful in differentiating between bacterial and viral pneumonia in children. We used proteomic methods to identify several potential salivary biomarkers to differentiate pneumonia from *Streptococcus pneumoniae* or influenza A virus infections in pediatric patients. Whether these salivary biomarkers can be used to distinguish other bacteria from viral pneumonia requires further verification.

## Figures and Tables

**Table 1:** The predicted pathway of more abundant salivary protein in *Streptococcus pneumoniae* group than Influenza A group.

Pathway Name	Enrichment *p*-Value
Valine, leucine and isoleucine biosynthesis	0.008463
Salivary secretion	0.016398
Complement and coagulation cascades	0.01818
Amoebiasis	0.023701
Lysosome	0.030797
2-Oxocarboxylic acid metabolism	0.038579
Pantothenate and CoA biosynthesis	0.039091
Glycosaminoglycan degradation	0.039091

**Table 2:** The predicted pathway of less abundant salivary protein in *Streptococcus pneumoniae* group than Influenza A group.

Pathway Name	Enrichment *p*-Value	Pathway Name	Enrichment *p*-Value
Vasopressin-regulated water reabsorption	0.000496	cAMP signaling pathway	0.013366
Amphetamine addiction	0.001445	Proteoglycans in cancer	0.015301
GABAergic synapse	0.001546	Nicotinate and nicotinamide metabolism	0.023197
Long-term potentiation	0.002042	Prion diseases	0.03016
Inflammatory mediator regulation of TRP channels	0.003325	Human papillomavirus infection	0.030801
Oocyte meiosis	0.003867	Hedgehog signaling pathway	0.032532
Platelet activation	0.004953	Endocrine and other factor-regulated calcium reabsorption	0.035263
Dopaminergic synapse	0.005182	Cocaine addiction	0.039439
Insulin signaling pathway	0.005399	Ovarian steroidogenesis	0.039982
Vascular smooth muscle contraction	0.005638	Vibrio cholerae infection	0.047745
Adrenergic signaling in cardiomyocytes	0.007017	Pyrimidine metabolism	0.047745
Oxytocin signaling pathway	0.007075	Regulation of lipolysis in adipocytes	0.049543

## Data Availability

The data can be requested by contacting the corresponding author via email upon publication.

## Supplementary Materials

The following supporting information can be downloaded at: https://www.mdpi.com/article/10.3390/diagnostics13081468/s1, Table S1. The differentially abundant proteins (determined as variation > 1.2-fold) between *Streptococcus pneumoniae* (P) group and Influenza A (F) group. Supplementary Figures S1–S10 showing the most significant differential pathways between *Streptococcus pneumoniae* group and Influenza A group. Figure S1: Valine, leucine and isoleucine biosynthesis; Figure S2: Salivary secretion; Figure S3: Complement and coagulation cascades; Figure S4: Amoebiasis; Figure S5: Lysosome; Figure S6: Vasopressin-regulated water reabsorption; Figure S7: Amphetamine addiction; Figure S8: GABAergic synapse; Figure S9: Long-term potentiation; Figure S10: Inflammatory mediator regulation of TRP channels.
