# Supplementary material for: Salivary Biomarkers to Differentiate between Streptococcus pneumoniae and Influenza A Virus-Related Pneumonia in Children
Source: Diagnostics (Basel). 2023 Apr 18;13(8):1468. doi: 10.3390/diagnostics13081468 (PMC10137754; doi:10.3390/diagnostics13081468)
Supplement: Supplementary file 1 [file diagnostics-13-01468-s001.zip › Supplementary Table S1.pdf]

Supplementary Table S1.

The differentially abundant proteins (determined as variation > 1.2-fold) between *Streptococcus pneumoniae* (P) group and Influenza A (F) group

	P/F		P/F		P/F		P/F
**HLA-A**	2.66	**PSMD3**	1.48	**DMKN**	1.35	**RAB15**	0.75
**SERPINA3**	2.20	**FLNB**	1.48	**BCAT2**	1.35	**PPP1CB**	0.75
**GGCT**	2.15	**DNER**	1.47	**TATDN1**	1.34	**EIF5A**	0.75
**TSPAN1**	1.87	**CRK**	1.47	**DNPH1**	1.33	**ANP32A**	0.73
**SFPQ**	1.83	**LPO**	1.46	**SERPINB9**	1.31	**STXBP2**	0.71
**COMT**	1.71	**ZCCHC6**	1.40	**MANSC1**	1.31	**PTBP1**	0.71
**CFD**	1.65	**CES1P1**	1.40	**SERPINB13**	1.29	**NT5C2**	0.70
**AMY1A**	1.58	**LY6D**	1.38	**SELENBP1**	1.29	**HNRNPH1**	0.65
**PRELP**	1.57	**SPRR1B**	1.38	**IGKV1D-16**	1.28	**PRKACB**	0.56
**SERPINB12**	1.56	**HSPE1**	1.37	**SERPINB2**	1.27	**ISG15**	0.54
**CES2**	1.54	**CTSA**	1.36	**LACRT**	0.78	**ARF3**	0.46
**SGSH**	1.52	**MAN1A1**	1.36	**NSF**	0.78		
