# Supplementary figures and images for: Salivary Biomarkers to Differentiate between Streptococcus pneumoniae and Influenza A Virus-Related Pneumonia in Children
Source: Diagnostics (Basel). 2023 Apr 18;13(8):1468. doi: 10.3390/diagnostics13081468 (PMC10137754; doi:10.3390/diagnostics13081468)

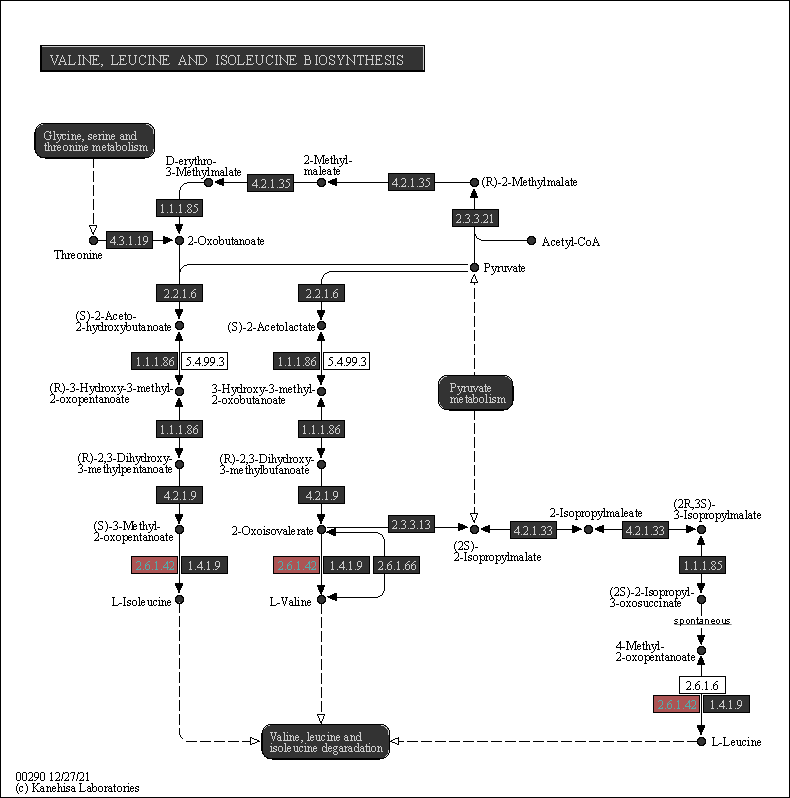

Supplement: Supplementary file 1 [file diagnostics-13-01468-s001.zip › Supplementary Figure S1.png]

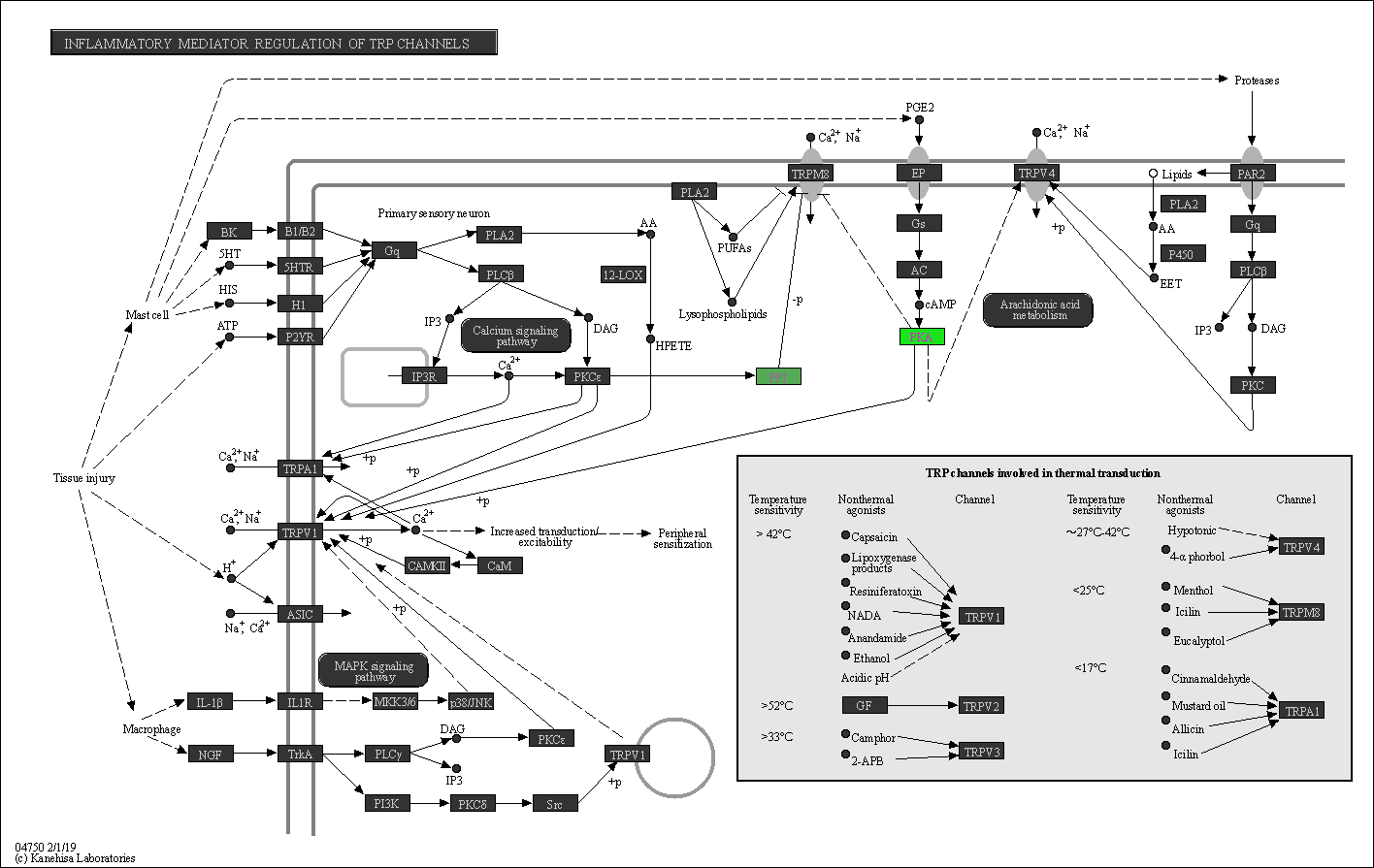

Supplement: Supplementary file 1 [file diagnostics-13-01468-s001.zip › Supplementary Figure S10.png]

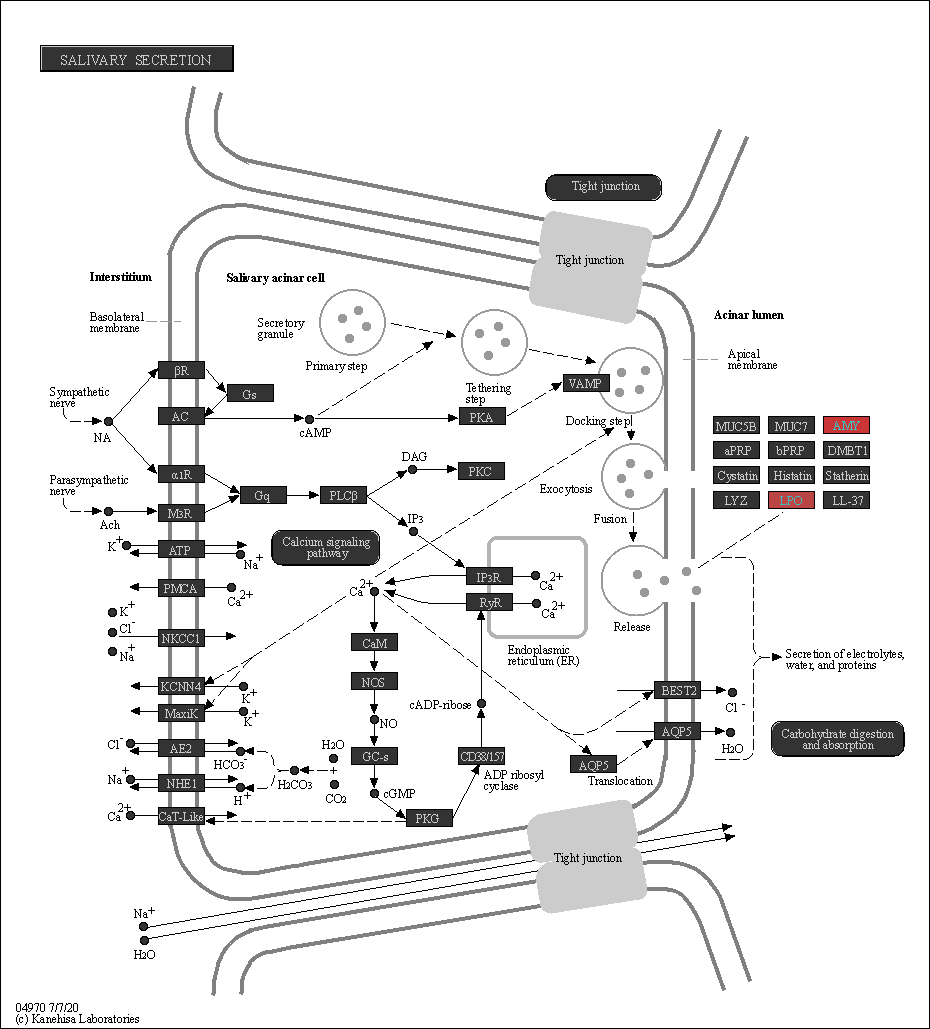

Supplement: Supplementary file 1 [file diagnostics-13-01468-s001.zip › Supplementary Figure S2.png]

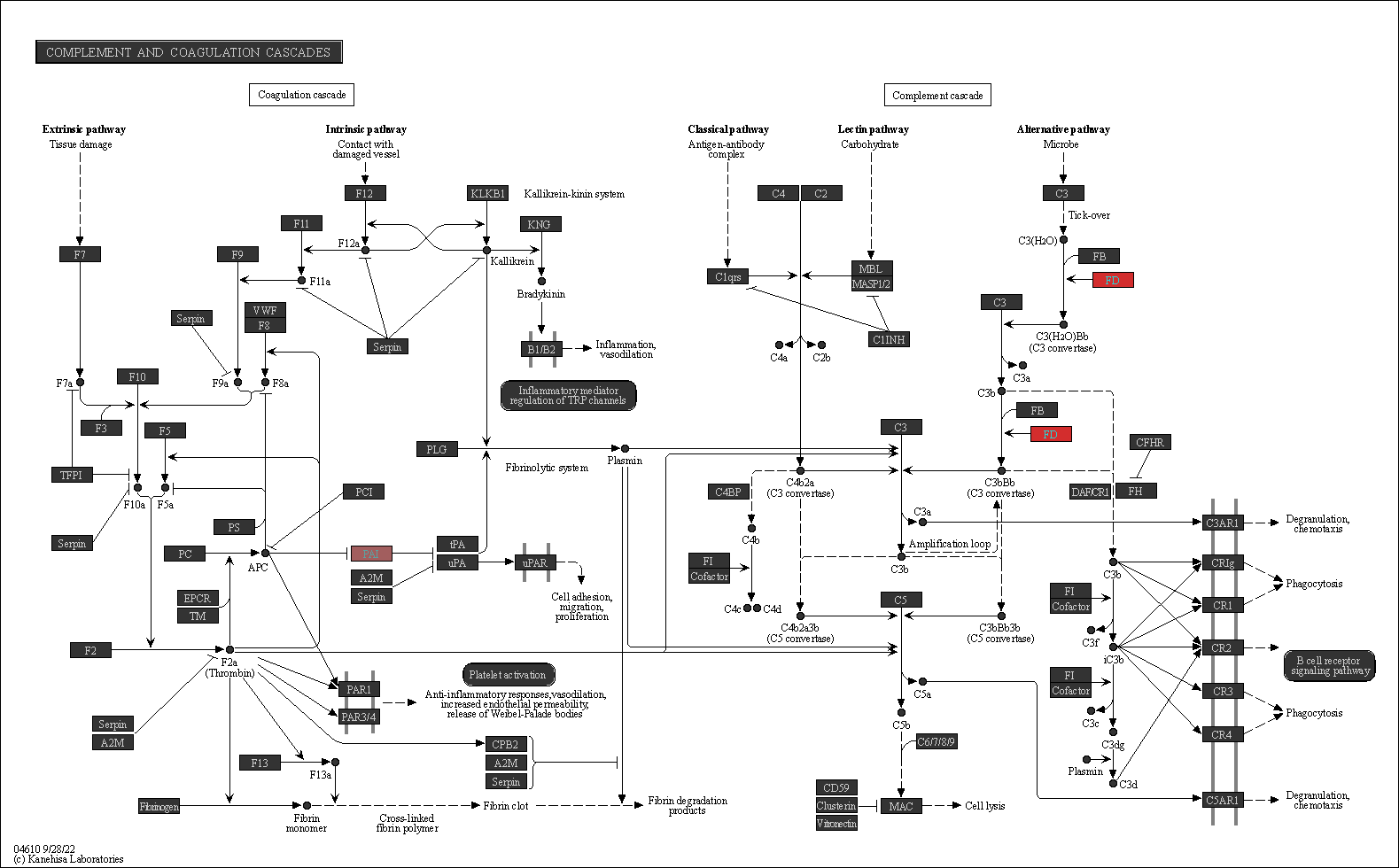

Supplement: Supplementary file 1 [file diagnostics-13-01468-s001.zip › Supplementary Figure S3.png]

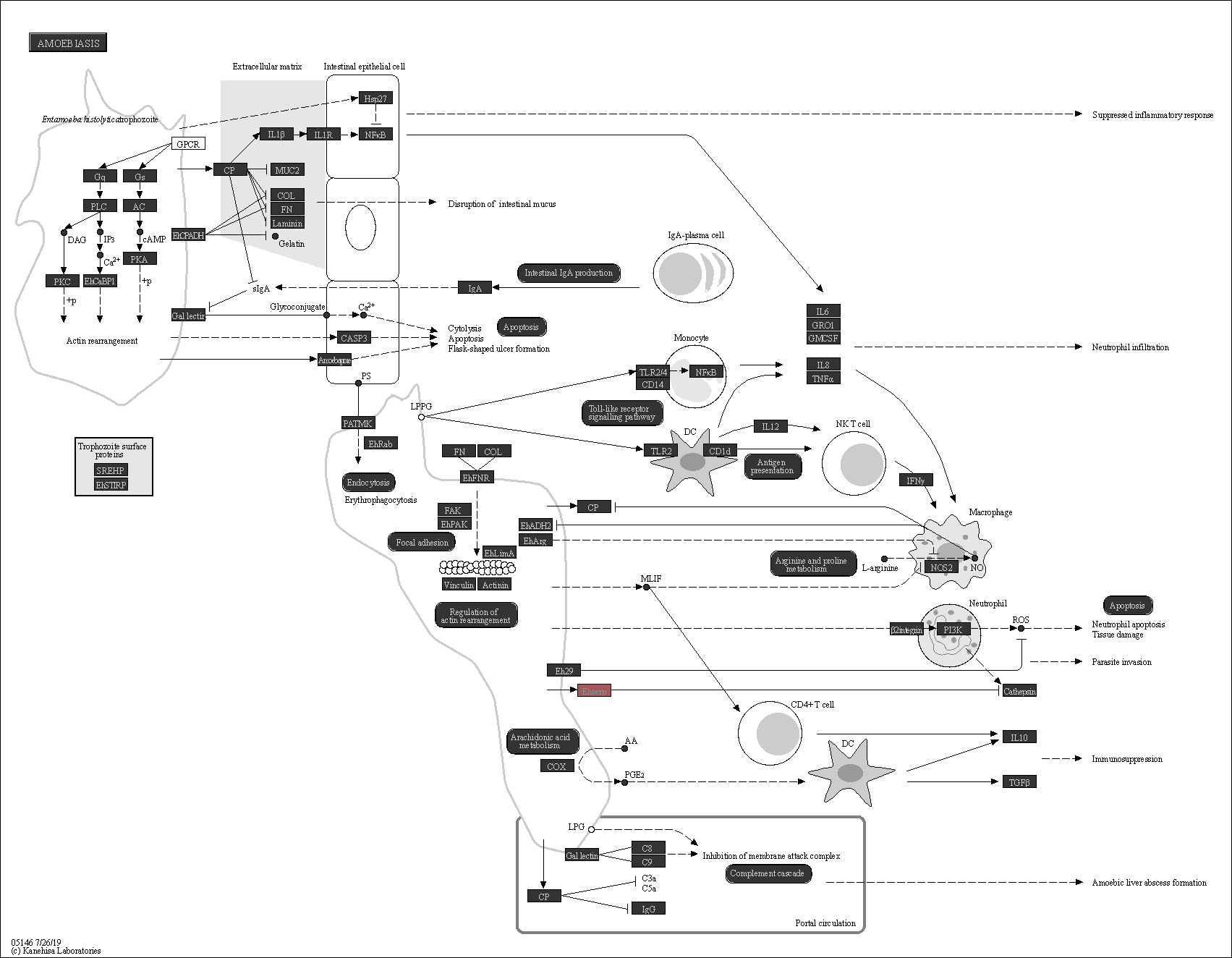

Supplement: Supplementary file 1 [file diagnostics-13-01468-s001.zip › Supplementary Figure S4.png]

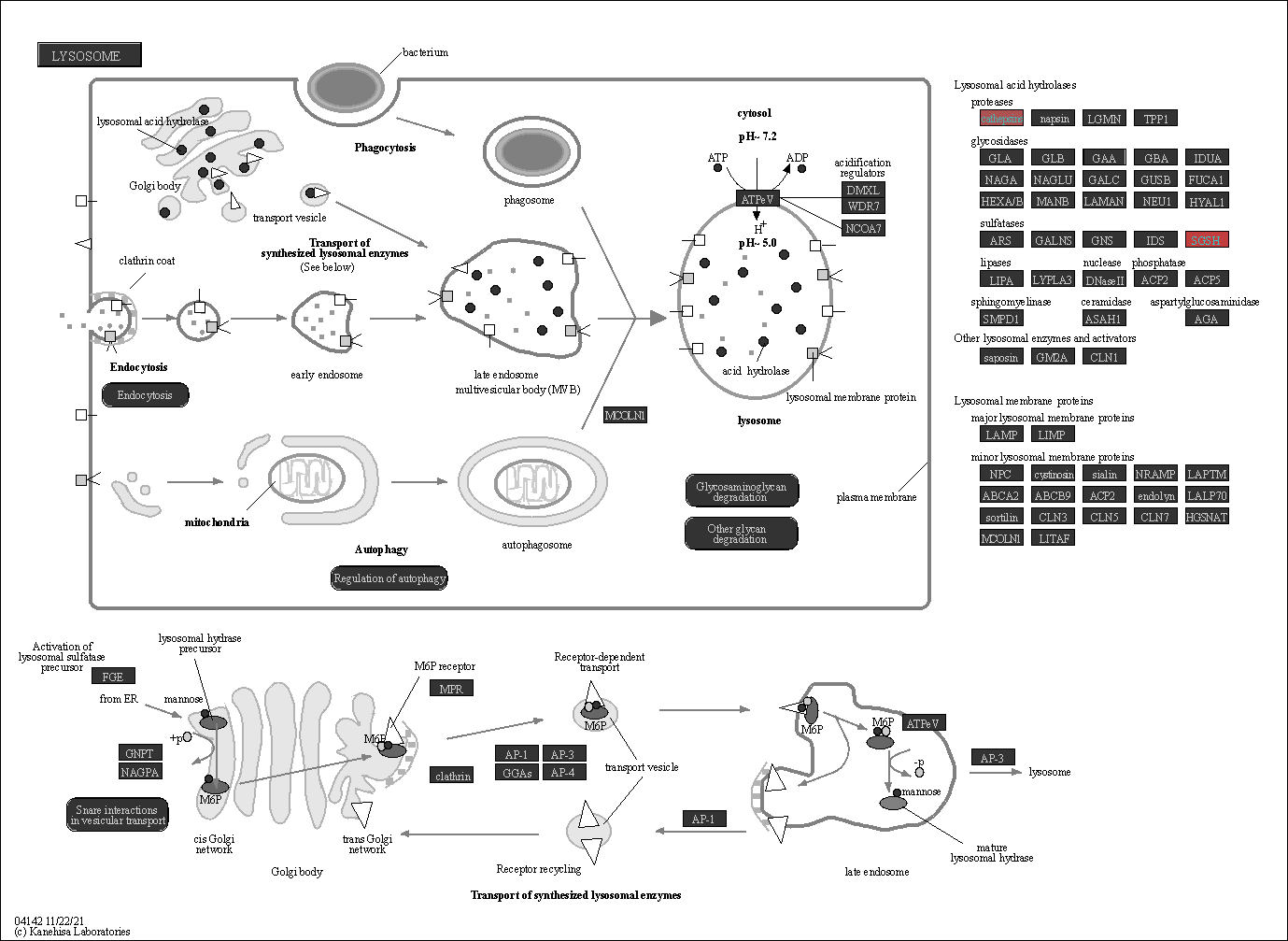

Supplement: Supplementary file 1 [file diagnostics-13-01468-s001.zip › Supplementary Figure S5.png]

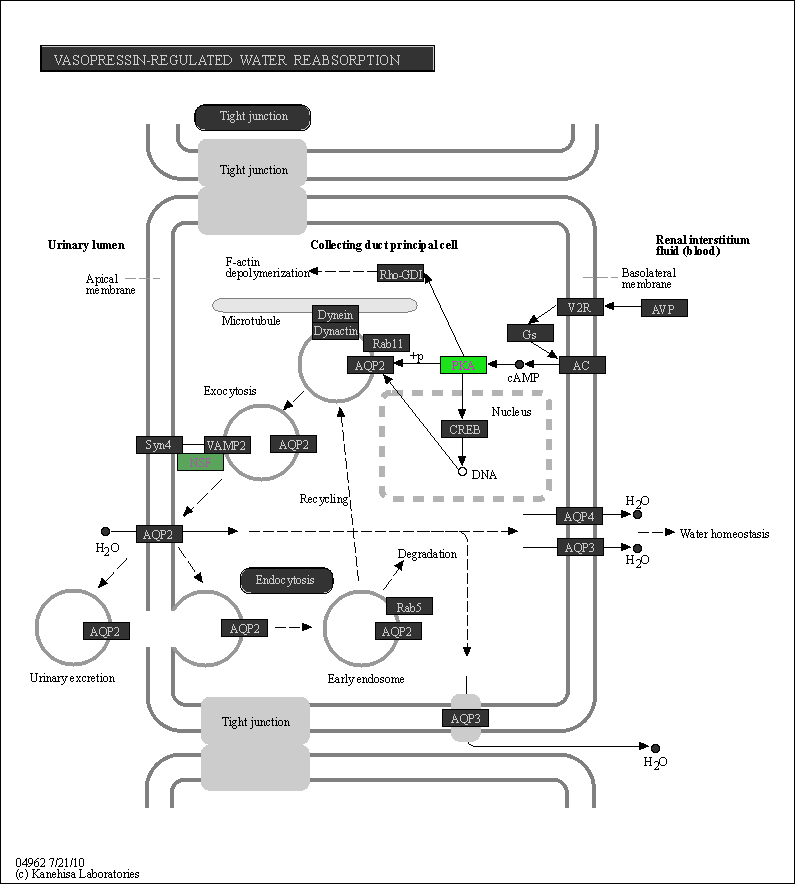

Supplement: Supplementary file 1 [file diagnostics-13-01468-s001.zip › Supplementary Figure S6.png]

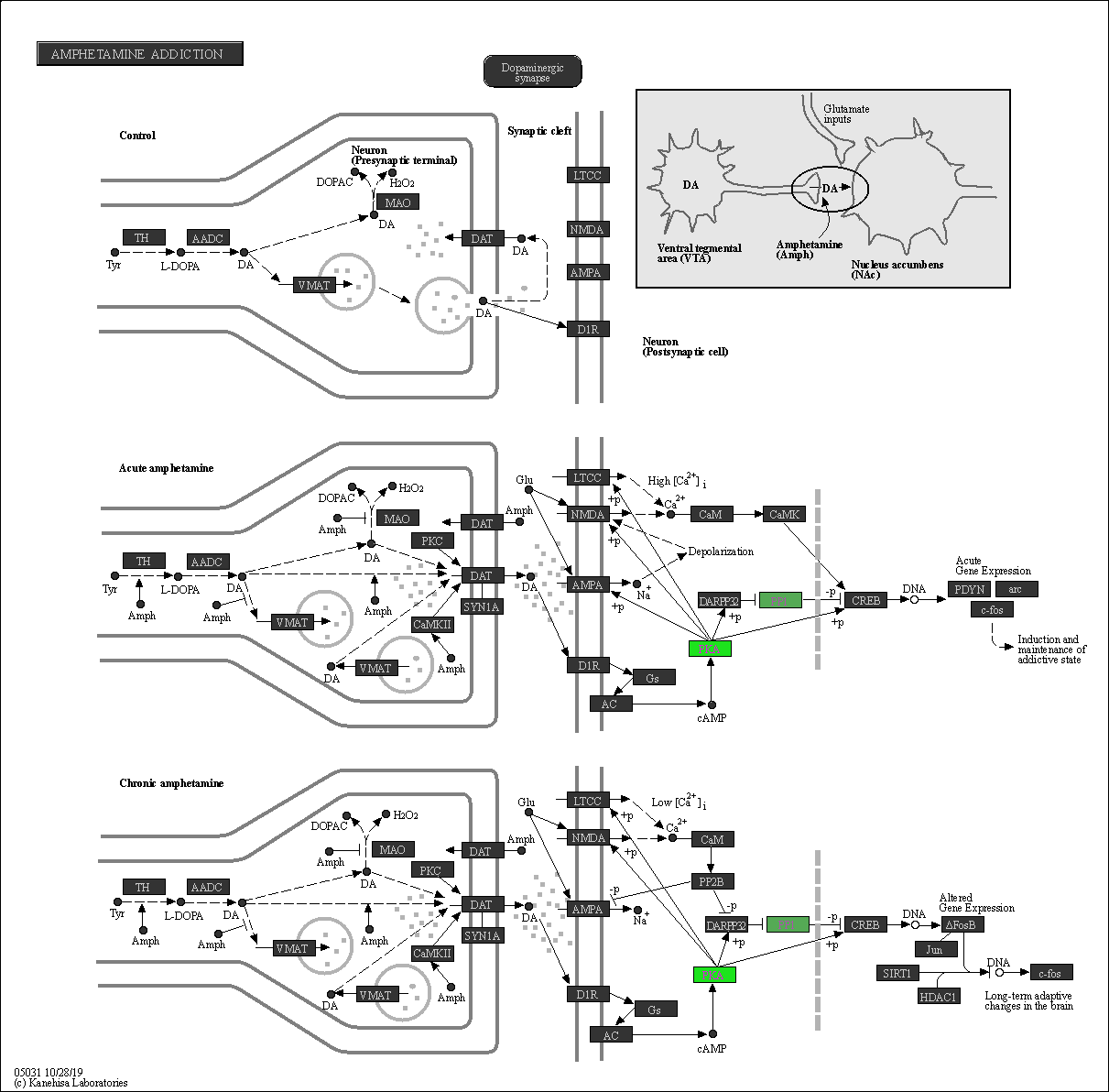

Supplement: Supplementary file 1 [file diagnostics-13-01468-s001.zip › Supplementary Figure S7.png]

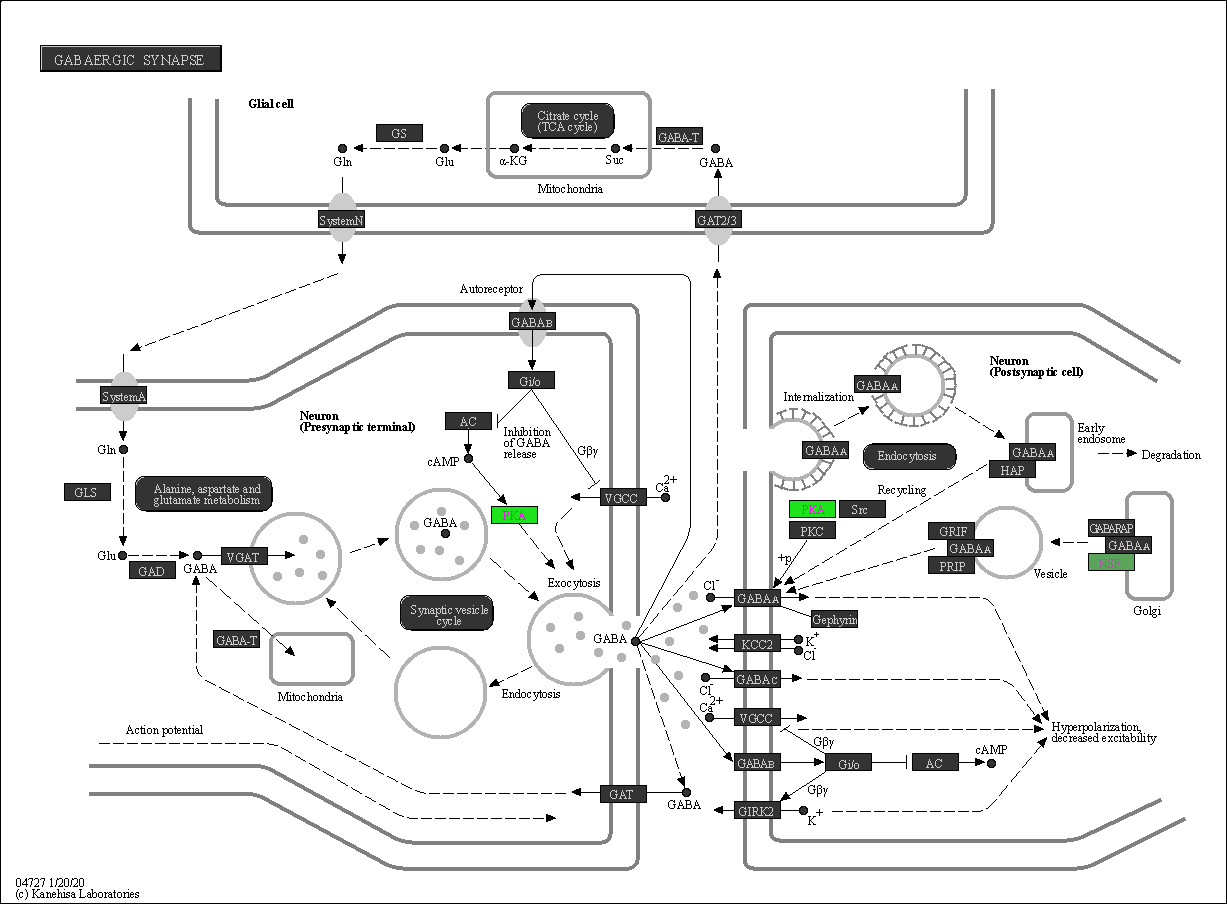

Supplement: Supplementary file 1 [file diagnostics-13-01468-s001.zip › Supplementary Figure S8.png]

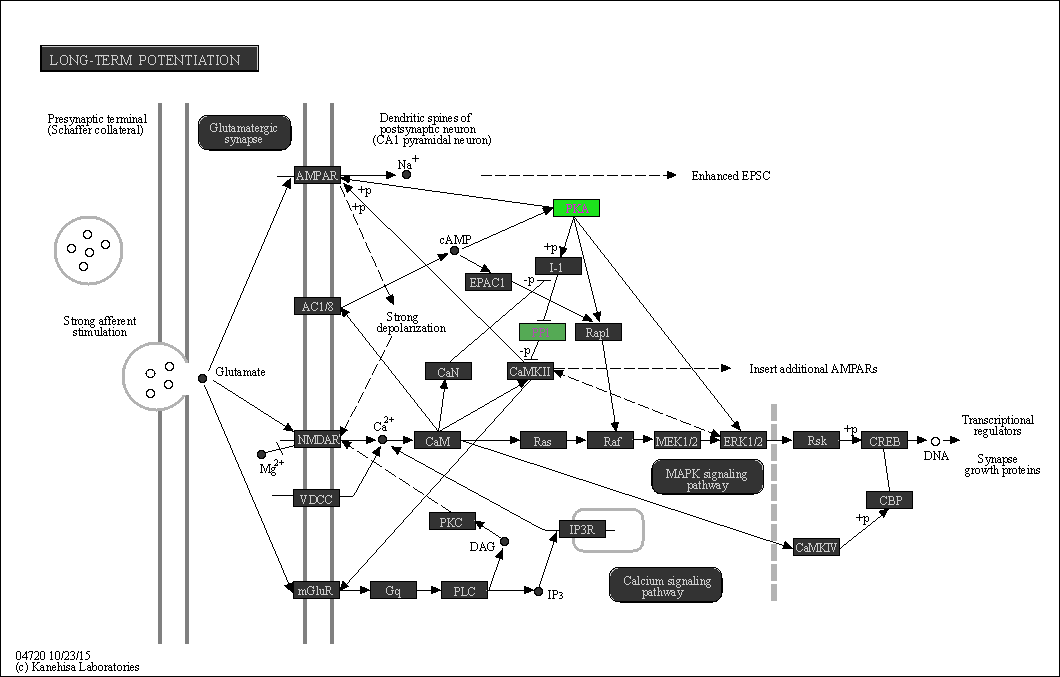

Supplement: Supplementary file 1 [file diagnostics-13-01468-s001.zip › Supplementary Figure S9.png]
